# PD206 Health Technology Assessment And Decision-Making During The COVID-19 Pandemic: Analysis Of Processes And Results From 25 Health Ministries

**DOI:** 10.1017/S0266462324004288

**Published:** 2025-01-07

**Authors:** Santiago Hasdeu, Jorgelina Alvarez, Anabel Beliera, Julian Sanchez Viamonte

## Abstract

**Introduction:**

The decision-making process in public administration can be analyzed from different perspectives. Evidence-based policies are not the only support for public policy design. High variability was observed regarding the health technologies used during pandemics. The processes and results of decision-making on health technologies in 25 health ministries in Argentina during the COVID-19 pandemic were analyzed.

**Methods:**

This retrospective study utilized triangulation of quantitative and qualitative methods. Information was retrieved for the years 2020 to 2021 through document review of official web pages, surveys, and interviews with decision-makers and advisors of the national ministry of health and 24 Argentinian provinces. Recommendations and reimbursement policies for seven health technologies were considered as tracers. Official health technology assessment (HTA) reports, laws, judicial rulings, journalistic news, and civil society actions on social networks and the internet regarding these technologies were analyzed. The ruling political party in each province was mapped, with the aim of exploring the political influences and intervening actors.

**Results:**

Contrary to World Health Organization (WHO) recommendations, ivermectin, inhaled ibuprofen, convalescent plasma, and equine serum were widely recommended and reimbursed outside a clinical trial context by most of the Argentinian ministries of health, leading to risks for patients and a huge opportunity cost. Health ministries with institutionalized HTA units had significantly higher adherence to WHO recommendations than other health ministries. Legislative and judicial powers influenced the use of health technologies through laws and judicial rulings. Researchers and civil society also influenced decision-makers. Partisan political issues did not fully explain the heterogeneity of the decisions made by the health ministries during the pandemic.

**Conclusions:**

The impact of HTA organizations and their technical reports was limited. Health Ministries with institutionalized HTA units were more likely to adhere to WHO recommendations. The influence of different technical and political criteria, power relations within and outside the administrations, the pharmaceutical industry and academics, the media, social pressure, judicial and legislative powers, and the political context strongly influenced decision-making.

